# Analysis of Graph Invariants in Functional Neocortical Circuitry Reveals Generalized Features Common to Three Areas of Sensory Cortex

**DOI:** 10.1371/journal.pcbi.1003710

**Published:** 2014-07-10

**Authors:** Suchin S. Gururangan, Alexander J. Sadovsky, Jason N. MacLean

**Affiliations:** 1 Department of Neurobiology, University of Chicago, Chicago, Illinois, United States of America; 2 Committee on Computational Neuroscience, University of Chicago, Chicago, Illinois, United States of America; Indiana University, United States of America

## Abstract

Correlations in local neocortical spiking activity can provide insight into the underlying organization of cortical microcircuitry. However, identifying structure in patterned multi-neuronal spiking remains a daunting task due to the high dimensionality of the activity. Using two-photon imaging, we monitored spontaneous circuit dynamics in large, densely sampled neuronal populations within slices of mouse primary auditory, somatosensory, and visual cortex. Using the lagged correlation of spiking activity between neurons, we generated functional wiring diagrams to gain insight into the underlying neocortical circuitry. By establishing the presence of graph invariants, which are label-independent characteristics common to all circuit topologies, our study revealed organizational features that generalized across functionally distinct cortical regions. Regardless of sensory area, random and 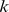-nearest neighbors null graphs failed to capture the structure of experimentally derived functional circuitry. These null models indicated that despite a bias in the data towards spatially proximal functional connections, functional circuit structure is best described by non-random and occasionally distal connections. Eigenvector centrality, which quantifies the importance of a neuron in the temporal flow of circuit activity, was highly related to feedforwardness in all functional circuits. The number of nodes participating in a functional circuit did not scale with the number of neurons imaged regardless of sensory area, indicating that circuit size is not tied to the sampling of neocortex. Local circuit flow comprehensively covered angular space regardless of the spatial scale that we tested, demonstrating that circuitry itself does not bias activity flow toward pia. Finally, analysis revealed that a minimal numerical sample size of neurons was necessary to capture at least 90 percent of functional circuit topology. These data and analyses indicated that functional circuitry exhibited rules of organization which generalized across three areas of sensory neocortex.

## Author Summary

Information in the brain is represented and processed by populations of interconnected neurons. However, there is a lack of a clear understanding of the structure and organization of circuit wiring, particularly at the mesoscale which spans multiple columns and layers. In this study, we sought to evaluate whether functional circuit architecture generalizes across the neocortex, testing the existence of a functional analogue to the neocortical microcircuit hypothesis. We analyzed the correlational structure of spontaneous circuit activations in primary auditory, somatosensory, and visual neocortex to generate functional topologies. In these graphs, neurons were represented as nodes, and time-lagged firing between neurons were directed edges. Edge weights reflected how many times the lagged firing occurred and was synonymous to the strength of the functional connection between two neurons. The presence of label-independent features, identified by investigating functional circuit topologies under a graph invariant framework, suggest that functionally distinct areas of the neocortex carry features of a generalized functional cortical circuit. Furthermore, our analyses show that the simultaneous recording of large sections of cortical circuitry is necessary to recognize these features and avoid undersampling errors.

## Introduction

Transmission and processing of information in the brain is in large part determined by the connectivity between neurons [1]. The neocortical microcircuit hypothesis states that the neocortex is composed of repeated elements of a generalized circuit that are tweaked for specialization in each area [2]. Supporting this hypothesis, local synaptic connectivity in the neocortex is non-random and is at least partly determined by neuron location and class [2]–[12]. These rules imply that there is a probabilistic or partially stereotyped wiring diagram. The extent to which these rules generalize across the neocortex, however, is unclear. Analysis of neocortical microcircuit spiking activity in different brain regions has revealed common dynamical features [12]–[15], suggesting that circuits may share similarities between regions. In this study, we use the spatiotemporal correlations of firing activity between neurons to generate functional wiring diagrams [15]–[19]. Modeling studies have shown a clear relationship between connectivity and neural firing [15], [20]–[24]. This suggests that we can gain insight into the underlying structure and organization of cortical circuitry by analyzing the emergent dynamics of large populations of neocortical neurons.

Here we employed high speed two-photon calcium imaging [25] to densely sample the spiking activity of up to 1126 neurons within a 1.1 mm diameter field of view, spanning multiple columns and layers in three different areas of the sensory neocortex. We then applied post-processing algorithms to detect spatiotemporal relationships between spiking neurons and modeled this activity as wiring diagrams, or graphs [15]. Graph theory is a useful technique to quantify network dynamics, and has been increasingly applied in the neural context to understand brain connectivity patterns [21], [26], [27]. One potential approach to identify invariant features of functional wiring diagrams within and between areas of cortex is to isolate graph isomorphisms. For example, the unlabeled graphs 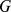 and 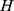 are isomorphic when any two nodes 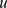 and 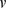 of 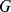 are connected in 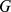 if and only if that connection exists in 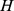. However, such an analysis currently remains intractable in graphs of sizes analyzed here, as the best known algorithm runs in polynomial time [28]. Perhaps more importantly, the organizational features of connectivity that have been described to date reflect probabilistic, rather than deterministic microcircuit architectures [5], [8], [9], making it unlikely that connectivity patterns in the brain are formally isomorphic. In order to test the postulate that the organization of functional circuitry generalizes across the neocortex, we instead applied functions that are invariant to labeling of the nodes of the graph. In other words, if A is the adjacency matrix describing graph 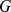, we wanted to describe the function 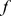 such that 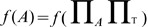, where 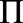 is the 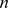
×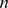 permutation matrix [29]. In the context of our study, we aimed to identify features of a neuronal circuit wiring diagram that are invariant to the particular identities of the neurons. Thus, we characterized each neuron only by the connections it had with other neurons. While neurons and activation patterns between animals and regions may vary in their individual details, these abstract, global characteristics of circuit structure stay constant, even following the relabeling of the neurons. By investigating label-independent features, called *graph invariants*, we hoped to disregard features of the functional circuit that may be susceptible to over-fitting, and focus on features that are stable across slices and areas of the neocortex. Many graph invariants have been previously described, such as maximum degree and MAXCUT value [29]. Some particularly useful invariants include the graph eigenvalues and eigenvectors [29], [30]. We apply these analyses to functional wiring diagrams generated from imaging data from three sensory neocortical areas to test the validity of a functional analogue to a generalized circuit architecture of the neocortex.

## Methods

### Ethics statement

All procedures were performed in accordance and approved by the Institutional Animal Care and Use Committee at the University of Chicago.

### Open source scientific software

To foster reproducibility and fast development of future work based upon these results, we have published functional graph analysis tools under an open source, GPLv3 license, available here: https://github.com/ssgrn/GraphInvariantsNeocortex.

### Data acquisition

#### Preparation of calcium dye-loaded slices

C57BL/6 mice of either sex on postnatal day 14–18 were anesthetized by intraperitoneal injection of ketamine-xylazine, rapidly decapitated, and had their brains removed and placed in oxygenated ice-cold cut artificial CSF (ACSF; contents contained the following, in mM: 3 KCl, 26 NaHCO_3_, 1NaH_2_PO_4_, 0.5 CaCl_2_, 3.5 MgSO_4_, 25 dextrose, and 123 sucrose). Coronal slices (500 µm thick) containing the sensory region of interest were cut perpendicular to the pial surface using a vibratome (VT1000S; Leica). To control for potential slice angle effects, slices at slight angles off the coronal axis were generated (500 µm A1/V1, 450 µm S1BF). Our data showed no significant effects that correlated with angle, and thus these datasets were pooled with their coronal counterparts [15]. Slices were placed in a 35°C oxygenated incubation fluid (Incu-ACSF; contents contained the following, in mM: 123 NaCl, 3 KCl, 26 NaHCO_3_, 1 NaH_2_PO_4_, 2 CaCl_2_, 6 MgSO_4_, and 25 dextrose) for 30 to 45 min. Calcium dye loading was then achieved by placing all slices into a small Petri dish containing ∼2 ml of Incu-ACSF, an aliquot of 50 µg Fura-2AM (Invitrogen) in 13 µl DMSO and 2 µl of Pluronic F-127 (Invitrogen) as previously described [25].

#### Calcium dye imaging

Experiments were performed in standard ACSF (contents contained the following, in mM: 123 NaCl, 3 KCl, 26 NaHCO_3_, 1 NaH_2_PO_4_, 2 CaCl_2_, 2 MgSO_4_, and 25 dextrose, which was continuously aerated with 95% O_2_, 5% CO_2_). Rapid whole-field imaging of Fura-2AM loaded neurons was achieved by taking multiple 5 min movies using the Heuristically Optimal Path Scanning technique and microscopy setup as previously described [25], allowing us to monitor action potential generation within individual neurons at scan speeds at least an order of magnitude greater than the traditional raster scan method. Cell contours were identified in an automated fashion as previously described [25]. Our dwell time parameter for each experiment was fixed at a value between 16 and 20 samples/cell/frame.

#### Laminar identification

We used biotinylated NeuN staining along with biocytin filled neurons which acted as fiduciary markers, in combination with measures of distance from pia and brightfield, NeuN, and two-photon cell density to identify lamina [15].

#### Spike and circuit event detection

Spikes were inferred from the fluorescence changes of individual neurons using a fast non-negative deconvolution algorithm that is a modified version of fast-oopsi [25], [31]. Spikes from each cell's calcium trace were then identified, and circuit events were defined as epochs in which the network of cells was active for at least 500 ms. The temporal precision of spike detection was dependent on the scan speed (∼125 Hz for 50 neurons and ∼8.5 Hz for 1,000 neurons) [25]. Greater than or equal to four events were necessary for a field of view to be included in our dataset.

#### Graph formation

Using rasters of spike trains inferred from calcium fluorescence changes, we generated circuit topologies corresponding to pairwise spiking correlations over all circuit events observed in a single field of view. Neurons were represented as nodes in each graph. Edges between nodes were directional and formed according to the following rule: neuron A was considered functionally connected to neuron B if neuron B fired in the subsequent frame. These edges were then weighted according to how many times this single frame lagged correlation occurred, normalized to the number of events in that field of view.

### Statistical analysis

All statistical analyses were performed with MATLAB (MathWorks). Unless otherwise noted, data are presented as mean ± SD. All 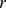 values in the text are in reference to the Pearson correlation computed with the command *corrcoef*. For nonparametric distribution comparison between the three sensory areas, the Kruskal-Wallis test (KW-test) was implemented via the *kruskalwallis* function. The nonparametric Komolgorov-Smirnov test (KS-test), noted at use, was used to compare fitted distributions to data. The Komolgorov-Smirnov test were implemented using the command *kstest2*. For tests of significance, 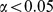 was used as the cutoff.

### Graph analysis

Algebraic connectivity and eigenvector centrality were computed using the MIT Toolbox for Network Analysis (http://strategic.mit.edu/downloads.php?page=matlab_networks). Graph figures were generated using the open source Python graph visualization tool NetworkX (http://networkx.github.io/). Circular variance was computed with the MATLAB Toolbox for Circular Statistics [32]. We compared our data to two null models: random topologies and 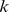-nearest neighbors topologies. Each random topology was formed by preserving the locations of neurons in a corresponding functional topology and then assigning a 0.5 probability of forming a directed edge between every neuron in the field of view. Each 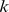-nearest neighbors topology was formed by preserving the locations of neurons in a corresponding functional topology and then forming a directed edge from neuron A to neuron B if neuron B was one of 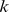-nearest neighbors of neuron A. In all analyses, we used 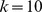.

## Results

### Formation of functional topology

**Figure 1:**
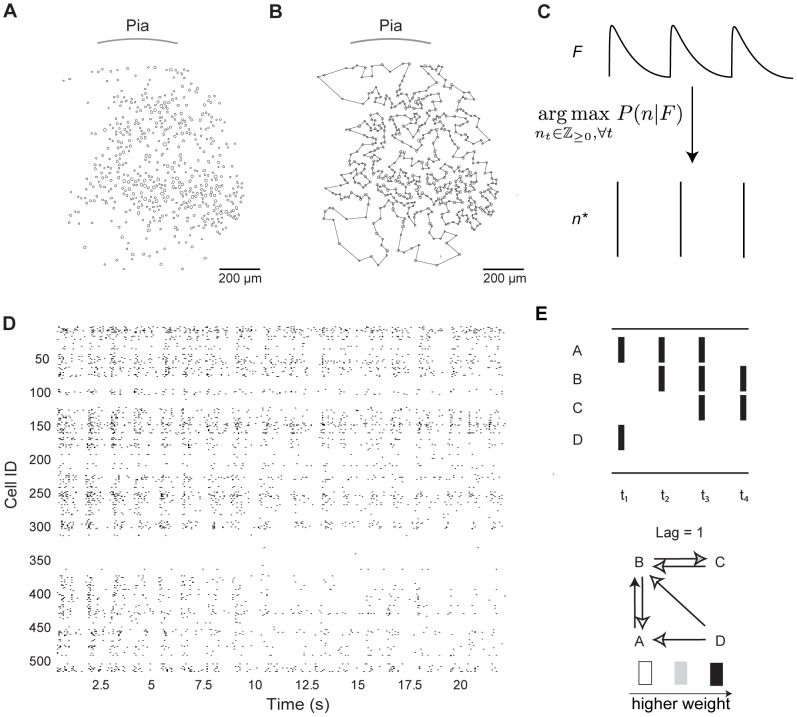
Sensory cortex exhibits spontaneous circuit activity. **A**) Automated cell detection from two-photon imaging of a slice of S1 cortex. **B**) Heuristically optimal path scan for two-photon imaging of same imaged field of view as A). **C**) Spike trains of each neuron were inferred from their calcium fluorescence signals. The deconvolution algorithm finds the maximum a-posteriori estimate of the probability of a spike train 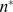 given calcium fluorescence signal 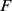. 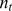 is the number of times the neuron spikes in frame 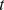. **D**) Examples of imaging network data as a raster. **E**) Cartoon example of the formation of a functional topology given a spike raster. Letters denote neuron label, 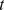 indicates frames. Directed functional connections are formed if one neuron fires one frame after another neuron. This connection is then weighted by how many times the single frame lagged correlation occurs.

To determine whether A1, S1, and V1 functional circuit wiring diagrams exhibited invariant features, we monitored neuronal activity in 43 slices from each region of the mouse neocortex (11 of A1, 21 of S1, and 11 of V1) using high speed multi-photon calcium imaging [15], [25], [31]. Spontaneous circuit activity requires intact excitatory amino acid transmission [15], [33], sufficient oxygenation [34] and corresponds to UP states within single neurons which comprise the functional circuit [15], [35]. Previous reports have found that spontaneous activity delineates all of the possible multi-neuronal patterns within a sampled population and that a sensory input activates only a subset of these patterns [14], [36]. By monitoring spontaneous activity in the imaged field of view, we hoped to maximize the number of pairwise correlations within the imaged populations. We imaged the flow of activity through large populations of neurons (A1: 595±101 cells, S1: 704±157 cells, V1: 734±129 cells) at the mesoscale in a two-dimensional circular imaging plane with a diameter of 1.1 mm that comprised multiple layers and columns with single-cell resolution (Figure 1A). We confirmed activity was not biased to any one lamina and that our sampling was uniform across our field of view, since the amount of activity observed across all circuit events did not differ between layers (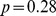, KW-test; see *Methods* for explanation of laminar identification). Because temporal resolution of multi-photon microscopy is compromised at these spatial scales, we used the heuristically optimized path scan technique [25] (Figure 1B), which allowed us to achieve fast frame rates (frame duration 86±17.7 ms) that did not differ between regions (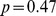, KW-test). We deconvolved calcium fluorescence changes of each detected neuron into spike trains (Figure 1C) [31] and generated rasters of spiking activity for the entire imaged population of neurons (Figure 1D). All regions of the sensory neocortex showed a common capacity for emergent, multi-neuronal patterned activity, characterized by discrete periods (>500 ms) of correlated action potential generation within subsets of neurons. Circuit events were separated by periods of quiescence and we refer to these distinct, clustered epochs of spontaneous action potentials as individual circuit events. The start and finish of a circuit event was easily resolvable because the field of view was either quiescent, corresponding to a DOWN state in a single neuron, or was active, corresponding to a UP state in a single neuron [35]. One circuit event lasted 1203±456 ms in A1,1568±885 ms in S1, and 1342±698 ms in V1. We imaged 82 total circuit events in A1, 268 total events in S1, and 104 total events in V1.

Using this data, we generated graphical abstractions, or circuit topologies, corresponding to functional activity over all circuit events observed in a single field of view. Neurons were represented as nodes in each graph. Edges between nodes were directional and formed according to the following rule: neuron A was considered functionally connected to neuron B if neuron B fired in the subsequent frame (Figure 1E). These edges were then weighted according to how many times this single frame lagged correlation occurred, normalized to the number of events in that field of view. Thus, stronger edge weights indicated reliable, correlated spiking, whereas weaker edge weights indicated unreliable, weakly correlated spiking (Figure 1E). The resultant graphs contained a large number of edges (median: 3.4×10^4^ functional connections, range: 4.2×10^5^ functional connections). Note that although a functional relationship between neurons increases the probability of them having a synaptic connection [18], [33], a linear relationship between each functional edge and a synaptic connection does not exist [16]. Rather, given our method of inference, the functional connectivity measure captured the flow of activity through the network during a circuit event.

### Neocortical functional circuits are characterized by invariant features

#### Functional circuitry is composed of non-random and occasionally distal connections

**Figure 2:**
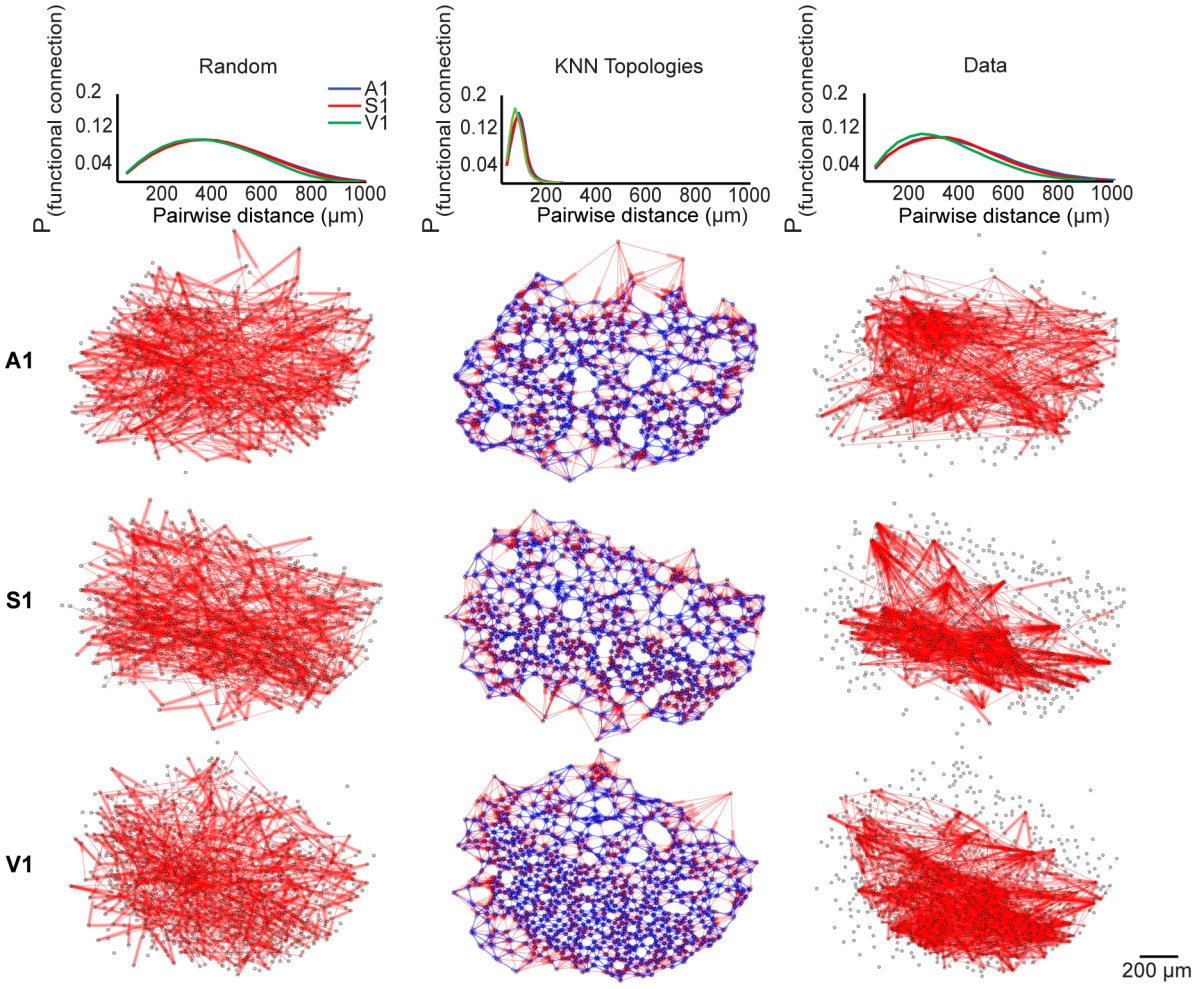
Functional topologies are composed of non-random, proximal and distal connectivity. The top row indicates distance-dependent probability distributions of functional connectivity. The other rows present representative examples of A1, S1, and V1 random graphs, 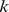-nearest neighbors topologies (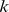 = 10), and functional topologies in data (labeled at the top). Probability distributions from individual slices did not differ from the mean distribution (Random graphs: 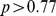, 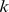-nearest neighbors graphs: 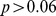, Data: 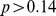; KS-test).

We found that most functional connections were locally organized and biased toward shorter pairwise distances, consistent with previous functional and anatomical studies [5], [15] (Figure 2, right column). To gain insight into the spatial dependency of functional circuit wiring, we compared functional topologies generated from the data with null models of varying spatial constraint. To this end, we generated a matched random and 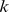-nearest neighbors null graph for each functional topology. The random and 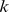-nearest neighbors topologies represented upper and lower bounds of spatial constraint, respectively. In each random topology, nodes were placed in the same locations as a corresponding functional topology, but each node had a fixed probability of functional connection (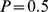) with any other node. We found that random topologies were spatially relaxed because their connections were not constrained to subsets or neighborhoods of nodes. Importantly, the spatial distribution of functional connections in random topologies was statistically indistinguishable from the long-tailed probability distribution of pairwise distances in the field of view (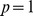; KS-test; Figure 2, left column). Thus, the random topologies still contained a distance dependence in its likelihood of a connection. The 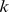-nearest neighbors topology was a null model consistent with previous anatomical studies that described synaptic connectivity in a nearest-neighbors paradigm [5]. In each 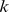-nearest neighbors topology, nodes were placed in the same locations as the corresponding functional topology, but neuron A was functionally connected to neuron B if and only if B was one of 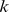-nearest neighbors of A (Figure 2, middle column). In this case, the probability of functional connection was heavily biased towards local neighborhoods, and thus the connections were spatially restricted. Because connections in random and 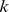-nearest neighbors topologies are non-specific beyond their spatial constraints, the poor quality of their fit to the data also provides insight into the prevalence of non-random functional connectivity that is not simply dependent on short distances.

Let 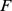 denote a functional connection derived from the data and 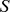 denote a functional connection in one of the corresponding nulls. To explore how well random and 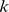-nearest neighbors topologies explained the data, we computed the following conditional probability distribution with respect to each model:
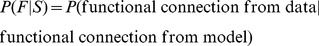
We expected the above expression to evaluate to 1 if there was a one-to-one relationship between edges in the 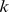-nearest neighbors or random topology and edges in the functional topology created from the data.

To calculate the above expression, we employed Bayes' rule:
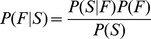
The 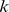-nearest neighbors topologies captured 12±9 percent of the total number of functional connections in the data, while the random topologies captured 9±8 percent of the total number of functional connections in the data. The amount of the data captured by the null models did not differ between regions (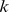-nearest neighbors: 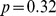, KW-test; random topologies: 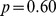, KW-test). Thus, functional topology is non-random, and connections that extend beyond local neighborhoods form a substantial portion of connections at the mesoscale.

To further characterize the distributed nature of functional topology, we next analyzed functional connections traveling between and within the lamina visible in our field of view (L1, L2/3, L4, and L5; see *Methods* for explanation of laminar identification). Due to relaxed spatial constraints and non-specific connectivity, Random topologies contained significantly more functional connections traveling between layers than within layers (between: 52±11 percent, within: 33±11 percent, 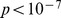). The difference in the number of functional connections traveling between and within lamina in random topologies was significant across areas (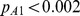, 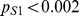, 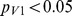). In contrast, 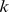-nearest neighbors topologies had significantly more functional connections traveling within layers than between layers (between: 11±3 percent, within: 82±11 percent, 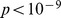). The difference in the number of functional connections traveling between and within lamina in 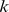-nearest neighbors topologies was significant across areas (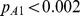, 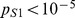, 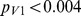). Functional topologies generated from the data had no significant difference between the number of functional connections traveling between layers and the number of those traveling within layers (between: 46±12 percent, within: 46±14 percent, p = 0.93, KW-test). Furthermore, the difference in the number of functional connections traveling between and within layers was insignificant across areas (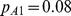, 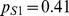, 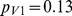).These analyses suggest that neocortical functional topologies consist of non-random, occasionally distal connections that, despite being skewed in probability toward local neighborhoods, are not solely governed by spatial proximity and are distributed across the field of view.

#### Neuronal influence in local functional circuitry is log-normally distributed

**Figure 3:**
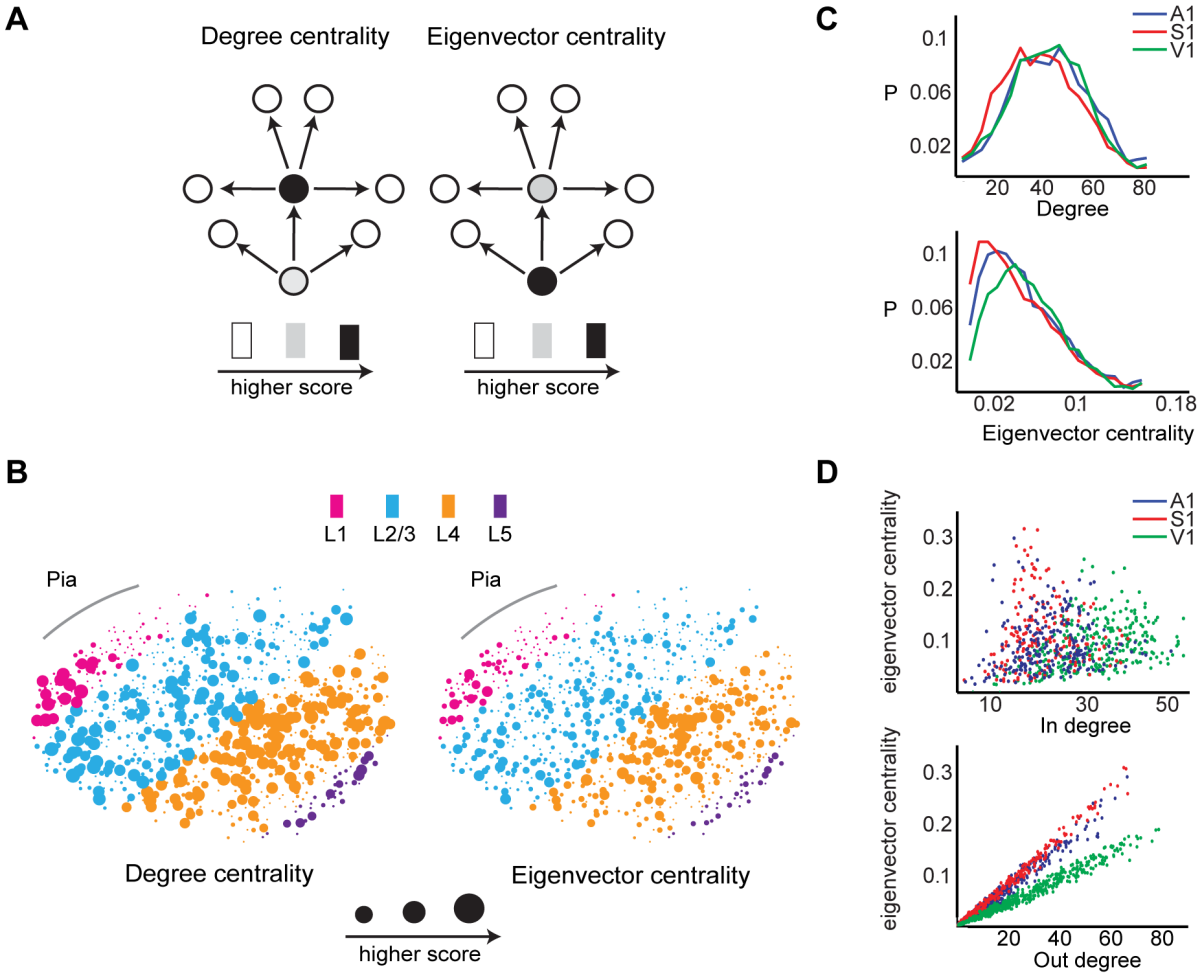
Hub neurons defined with eigenvector centrality. **A**) Illustrative example showing differences between degree and eigenvector centrality measures. Neurons that are more influential in driving local circuit flow are scored higher in the eigenvector centrality measure, whereas neurons that have the largest number of connections are scored higher in the degree measure. **B**) Degree and eigenvector centrality measures projected onto the same labeled A1 slice. Larger dots indicate neurons with higher score. **C**) Mean probability distributions of degree and eigenvector centrality in A1, S1, and V1. Distributions from individual slices did not differ from the mean distribution (Degree centrality: 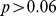; Eigenvector centrality: 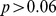; KS-test). **D**) Scatter plots of eigenvector centrality vs in degree (top) and vs. out degree (bottom) for representative examples in A1, S1, and V1.

Neuronal networks have been found to contain neurons which are connected to large numbers of other cells, called hubs [15], [21], [37], [38]. Traditional approaches characterized a hub as having a large degree that is multiple standard deviations from a network's norm [39]. However, this metric of degree centrality fails to fully capture the influence of a node in a network. To identify network hubs that focused on functional information flow, we utilized the eigenvector centrality measure of node influence. Let 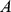 denote an 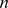
×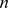 adjacency matrix. Then the eigenvector centrality 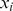 of node 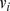 is defined as the 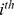 entry in the normalized eigenvector corresponding to the largest eigenvalue of 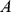.
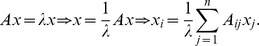
The above implies 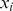 is a linear combination of centrality scores of all nodes connected to 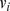; a node that has a high eigenvector score is connected to nodes that are also high scorers. Uniqueness of the eigenvector associated with the largest eigenvalue 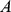 is ensured by the *Perron-Frobenius Theorem*, which states that any positive definite square matrix has a unique largest real eigenvector with strictly positive components [40]. The difference between eigenvector centrality and degree measures is revealed in the following example. Let one neuron project an edge to another neuron, which in turn projects to ten neurons. The first neuron in this chain would be assigned a degree of 1, and thus would be considered an insignificant actor in the circuit under degree centrality. However, under eigenvector centrality, each neuron's score is a linear combination of all other neurons' scores. Eigenvector centrality would assign the first neuron a high score as it is considered to be the most influential driver of local activity (Figure 3A). Projections of both measures onto an imaged field of view qualitatively revealed differences in the contour distributions of assigned scores by degree and eigenvector centrality (Figure 3B). We calculated the distribution of eigenvector centrality and degree scores, and found that the former fit to a log-normal distribution in all three areas of the sensory neocortex (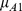 = −3.88±0.07, 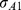 = 1.01±0.05, 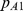 = 0.13; 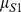 = −4.01±0.07, 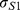 = 1.05±0.05, 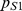 = 0.08; 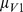 = −4.08±0.08, 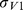 = 1.21±0.06, 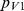 = 0.13; KS-test; Figure 3C), and that the latter fit to a normal distribution in all three areas of the sensory neocortex (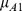 = 0.05±0.002, 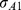 = 0.03±0.001, 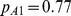; 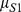 = 0.04±0.002, 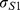 = 0.03±0.002, 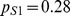; 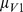 = 0.05±0.002, 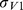 = 0.03±0.002, 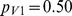; KS-test; Figure 3C). Interestingly, we found that eigenvector centrality scores were not correlated with in-degree (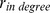 = 0.14±0.24, 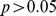; Pearson correlation; Figure 3D), but were highly correlated with out-degree (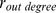 = 0.98±0.04, 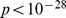; Pearson correlation; Figure 3D). The strength of the correlation did not differ between regions (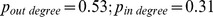; KW-Test). The tight relationship between eigenvector centrality and out degree implies that the influence of neuron in its local circuit is highly related to its feedfowardness. Thus, it is interesting that V1's eigenvector centrality distribution is translated to greater eigenvector centrality scores relative to A1 and S1, given V1's higher propensity for feedforward activity (Figure 3C) [41], [42].

#### Functional circuit topologies are connected

A graph is connected if there exists a sequence of edges from any node to any other node. To quantify the connectedness of neocortical functional topologies, we employed the following theorem:

**Theorem:** Let the undirected graph 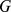 be specified by an adjacency matrix 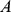 and have a degree matrix 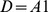, where **1** is the column vector of all 1 s. Let the Laplacian 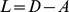 have eigenvalues 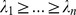. 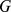 is connected if and only if 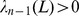. 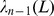 denotes the algebraic connectivity of 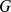. (For proof, see [30])

**Figure 4:**
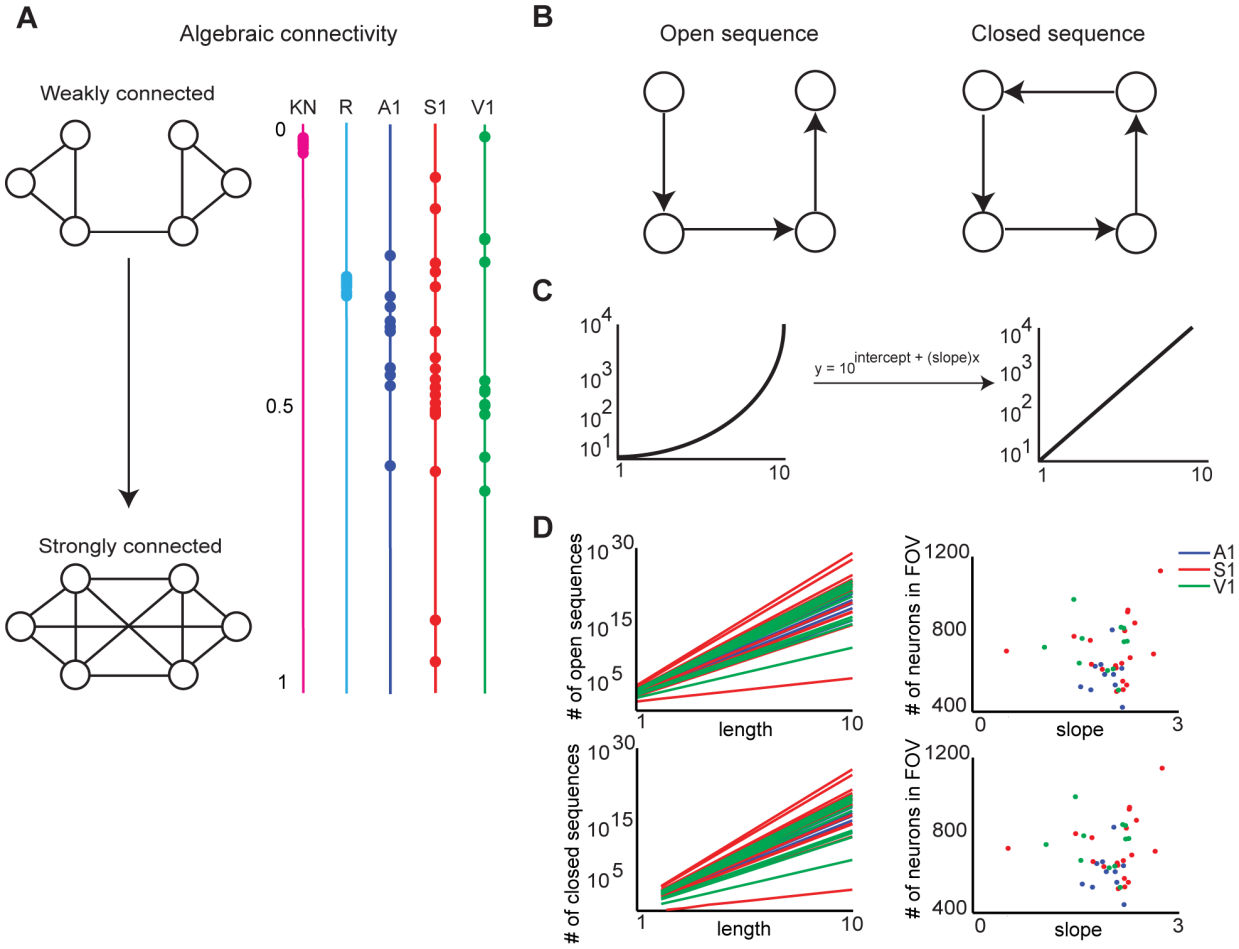
Functional topologies are connected and their size is independent of the number of neurons sampled. **A**) Algebraic connectivity (normalized by number of nodes in the graph) of functional topologies in A1, S1, and V1. An algebraic connectivity closer to 0 indicates a weakly connected graph, whereas an algebraic connectivity closer to 1 indicates a strongly connected graph. **B**) Sequences are walks on adjacent nodes. Open sequences start and end on different nodes, while closed sequences start and end on the same node. **C**) Linearization of exponential plots by plotting y-axis values in log-scale. **D**) Left column: Linearized plot of number of open and closed sequences of given lengths in functional topologies generated from data in A1, S1, and V1. Right column: Scatter plots of slopes of growth curves on left column vs. the number of neurons in the corresponding fields of view.

The larger the algebraic connectivity is, the more strongly connected the graph is. An algebraic connectivity close to zero indicates a graph that is highly modular and susceptible to attack, which makes connectedness a prime topological metric for defining robust networks [43]–[45]. We assessed the connectedness of the functional topology by first transforming all directed edges to undirected ones, as this is required for the theorem to be applicable. We therefore lost information on circuit flow provided by directed edges, but preserved information on the abstract structural features of the topology, like the general reachability a neuron in the circuit. We then computed the second smallest eigenvalue of the Laplacian of the resulting adjacency matrix, normalized by the number of nodes in the graph. We found that functional topologies in each sensory area were connected (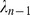 = 0.385±0.17; Figure 4A) and that the amount of connectivity did not differ between regions (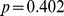; KW-test). These values significantly differed from the moderately connected random topologies (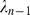 = 0.24±0.006; 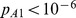, 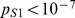, 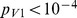; KW-test) and the weakly connected 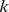-nearest neighbors topologies (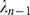 = 0.02±0.004; 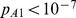, 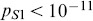, 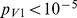; KW-test). This analysis suggests that an arbitrary path from any neuron to every other neuron is present in functional circuit topologies. Interestingly, the variance of the algebraic connectivities of the models was much smaller than those of the data. The greater variance of algebraic connectivity present in the data might emerge from specific patterns of functional connectivity that are absent in the non-specific random and 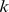-nearest neighbors topologies.

#### The size of functional circuit topologies does not scale with the number of neurons in the field of view

Sequences of neuronal activations in the neocortex likely represents a neural syntax that encodes external stimuli [46]. Since each directed edge in a functional topology represents a sequential activation of two neurons, each activation sequence can be defined as a walk, or a sequence of visitations to adjacent nodes, in the functional topology. Because spontaneous activations delineate all possible multi-neuronal patterns within a sampled population [14], [36], we quantified the number of possible activation sequences of a given length in functional topologies generated from spontaneous activity. To compute this metric, we employed the following theorem:

**Theorem:** Let the graph 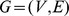 be specified by an adjacency matrix 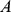. For any 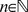, the 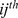 entry of the matrix 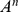 is equal to the number of walks from 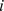 to 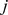 in 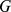 of path length 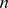.

This theorem can be proved through induction; we use the facts that each edge in the graph is unique, and that to form a walk of length 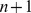 from vertex 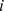 to 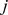, one must first have a walk of length 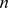 from vertex 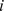 to 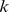, and then a walk of length 1 from vertex 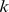 to 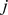. Note that the number of open sequences, walks that do not have equal starting and ending nodes, is the sum of the upper and lower triangular matrices of 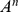 (Figure 4B). In addition, the number of closed sequences, walks that have equal starting and ending nodes, is the trace of 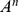 (Figure 4B). Open sequences may relate to feedforward activity, while closed sequences may relate to recurrent activity [15], [42]. In all analyses, we computed the number of open sequences of path lengths 1 to 10 and the number of closed sequences of path lengths 2 to 10, since a closed sequence of length 1 does not exist. We found that the number of possible open sequences and closed sequences, as a function of path length, were perfectly fit by exponential functions across all functional circuits (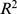 = 1.00±0.00). This exponential growth reflected the combinatorial explosion of possible sequences of larger lengths, as the graphs analyzed contained a large number of nodes and edges.

Next, we computed the ratio of the number of open sequences to the number of closed sequences in each graph, excluding sequences of length 1. We refer to this ratio as the *O-C ratio*. We found that across all path lengths analyzed, there were 217±47 open sequences for every closed sequence in A1, 293±151 open sequences for every closed sequence in S1, and 339±67 open sequences for every closed sequence in V1. The higher O-C ratio in V1 likely supports the postulate that the region has a greater propensity towards feedforwardness [37], [42].

The O-C ratio did not differ between path lengths (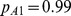, 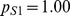, 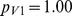; KW-test), suggesting that while the *raw number* of open and closed sequences grows exponentially as a function of path length, the *ratio* of open to closed sequences stays constant. In contrast, the O-C ratio in random topologies increased 2-fold from length 2 to length 3 (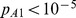, 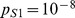, 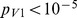; KW-test), and stayed constant for larger path lengths (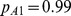, 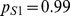, 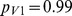; KW-test). Further analysis showed that random topologies had a far greater percent of possible reciprocal connections (closed sequences of length 2) than functional topologies generated from the data (random: 24.9±0.04 percent; data: 8.4±8.2 percent).The greater prevalence of reciprocal connections in random topologies likely results in the smaller O-C ratio at length 2.

Because the number of open and closed sequences as a function of path length grew exponentially, we could linearize the curves by transforming them into log-scale (Figure 4C). Linearization allowed us to use slope as a feature of how the number of sequences varied with path length. We found that the distribution of slopes did not differ between open and closed sequence growth curves for all functional topologies generated from the data (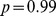; KW-test). This finding confirmed the invariance of the O-C ratio to path length in the data.

We found that the slope of a sequence growth curve was strongly correlated with the number of functional connections in the corresponding topology (Open and closed sequences: 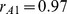, 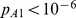; 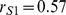, 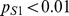; 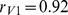, 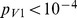; Pearson correlation). This finding prompted us to characterize how the number of sequences in a functional topology varied with the number of neurons in the field of view. We hypothesized that random topologies were greedy: the more nodes in the field of view, the more activation sequences would be possible, because every node in the random topology has a 0.5 probability of being connected to any other node. Thus, the size of the random topology would scale with the number of nodes in the field of view. Supporting this hypothesis, we found that the slope of sequence growth curves for random graphs were strongly correlated with the number of nodes in the corresponding random graph in all regions (Open and closed sequences: 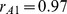, 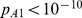; 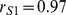, 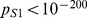; 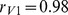, 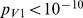; Pearsons linear correlation). In contrast, we found that the slopes of sequence growth curves in the data were uncorrelated with the number of neurons in the corresponding functional topologies in all regions (Open and closed sequences: 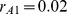, 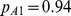; 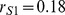, 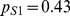; 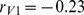, 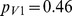; Pearson correlation; Figure 4D). This finding suggests that the size of functional connectivity does not scale with the number of neurons in the field of view, and that only a subset of neurons in the field of view are recruited during any one circuit event. These analyses further support the postulate of specificity in functional connectivity, and suggest that the lack of strong positive correlation between the slope of the sequence growth curve and number of neurons in the field of view is inherent to the functional connectivity patterns of these regions.

### Local circuit flow covers entire angular space

There is an ongoing debate on whether the cortical column, which is oriented perpendicular to pia, regulates and shapes the flow of information in sensory cortices [47]. Coronal slices allowed us to image activity patterns with near simultaneity across all lamina. Using this data, we assessed directional flow in functional graphs by computing the angle and distance between the source and destination of directed functional connections relative to the orientation of pia. *Flow maps* are plots that capture direction of circuit flow with points scattered at a radius 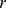 and angle 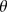 about the origin. 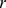 represents the distance of the functional connection from the source to the sink, and 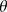 represents the angle between the source and the sink.

We measured the amount of angular clustering of activity flow in sensory areas by computing the circular variance of functional connections. The clustering of points at a particular angle indicates stereotypy of functional flow across events in a neighborhood of the functional topology. We calculated the amount of angular clustering by computing the circular variance of the set of 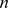 points.

**Figure 5:**
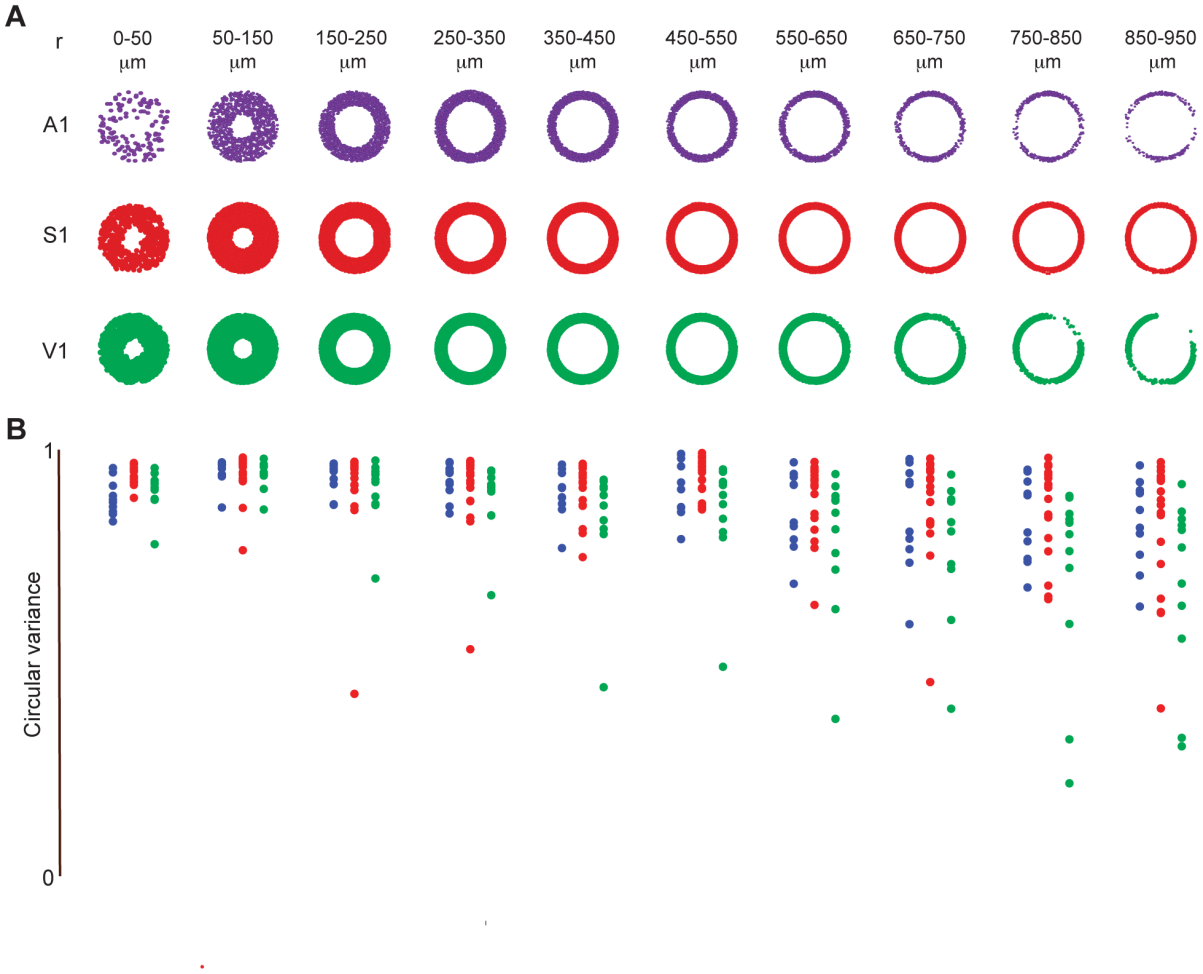
Circuit activity flow uniformly covers angular space. **A**) Representative examples of flow maps at multiple pairwise spanning distances between two nodes in A1, S1, and V1. Each point indicates the angle between the source and sink of a functional connection, relative to the orientation of pia. **B**) Circular variance of flow maps in A1, S1, and V1.

Circular variance is defined as:
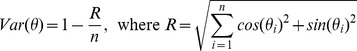
The value of the circular variance varies from 0 to 1; the lower the value, the tighter the clustering of points about a single mean angle. In functional circuit topologies from all three areas of the sensory neocortex, flow covered the entire angular space, regardless of the pairwise distance, or radius, spanned by the functional connection (Figure 5A). We found that the spread of circular variance increased for functional connections which spanned the largest distances, most likely due boundaries imposed by pia, internal capsule, or field of view (Figure 5B). Thus we did not find a canonical circuit flow in spontaneous cortical activity regardless of sensory area.

### Large fields of view are necessary to investigate functional topologies

**Figure 6:**
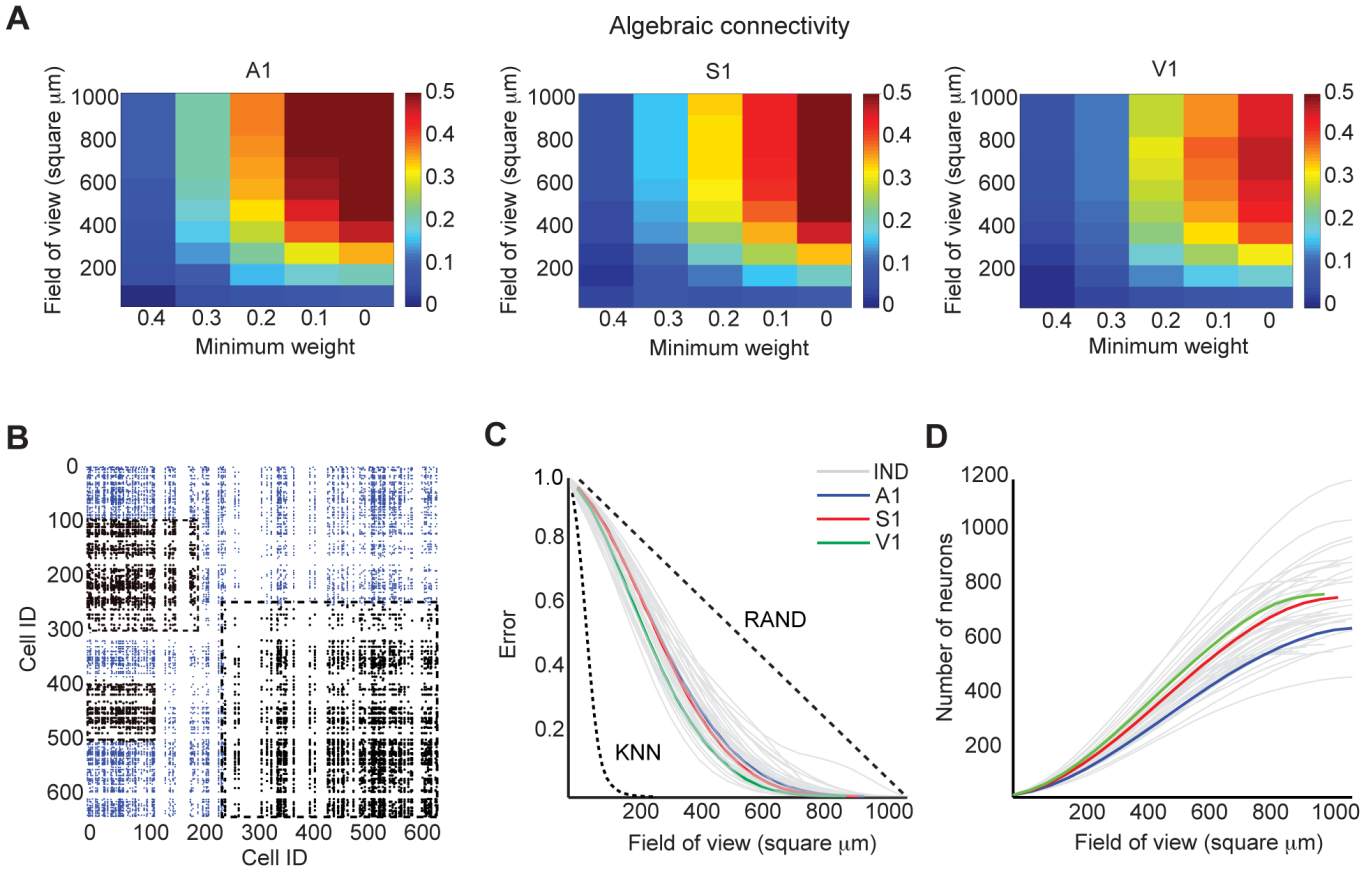
A minimum field of view is necessary to capture functional topology. **A**) Mean algebraic connectivity (normalized by number of nodes in the graph) given a field of view size (maximum pairwise distance) and minimum weight in A1, S1, and V1. **B**) Representative example (in A1) of how field of view (FOV) influences the number of functional connections captured. Dashed boxes are arbitrarily located on the connectivity matrix and are used to illustrate how different sized FOVs determine the functional connections (black) that can be resolved within that imaged area. Blue ticks indicate functional connections we cannot see with given FOV. **C**) FOV error of functional topologies generated from data (gray: data from individual slices (IND); colored: mean of data across slices from each sensory area (A1, S1, V1)). Dashed lines correspond to global mean FOV errors in 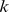-nearest neighbors topologies (KNN) and graphs with uniform probability of functional connection given pairwise distance (RAND). FOV errors from individual null topologies did not differ from the global mean FOV error (KNN: 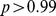; RAND: 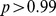; KS-test). **D**) Corresponding density of neurons given FOV size (gray: data from individual slices (IND); colored: mean of data across slices from each sensory area (A1, S1, V1)).

The highly distributed nature of functional topologies suggested that large fields of view are necessary to fully capture invariant features of functional topology. We sought to confirm this hypothesis by examining the spatial dependency of connectedness in functional topologies. Connectedness in the context of an imaged field of view can be described as an aperture problem: large interlinked networks look like disjoint groups of interacting cells if viewed only in small parts, while viewing the entire network at once reveals one giant component. For efficient computation in our graph invariant framework, we examined this problem in the following way: disjoint modules of network activity could be characterized as a weakly connected functional topology with a small algebraic connectivity. We explored how algebraic connectivity of the functional topology was modulated by two variables: minimum weight and field of view size. Because edge weight corresponds to the reliability of an observation of a spike correlation, thresholding minimum weight in a functional topology pruned its weaker edges. We defined field of view size as the maximum pairwise distance between any two neurons investigated. Together, these variables represented spatial and sampling bias during experiments. We found that the algebraic connectivity of functional topologies followed similar trajectories in all three sensory areas: smaller fields of view and the exclusion of the weakest functional connections resulted in weakly connected graphs (Figure 6A). Taken together, these data suggest that one must employ large fields of view and low edge weight thresholds to capture an independent functional circuit.

Interestingly, we found a field of view size in each sensory area at which the algebraic connectivity seemed to reach capacity or asymptote; above this distance, larger fields of view did not result in significantly increased connectivity. This finding suggested that a subsample size less than 1.1 square mm would capture a complete functional circuit topology. To further understand the interplay between experimental field of view and the topology of the functional circuits, we specified a general model of *Field of View (FOV) Error*, or how well a functional topology is captured as a function of field of view size (Figure 6B).

FOV error varies with the distribution of functional connections inherent to each neocortical region (Figure 2, right column). Formally, let 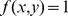 denote the existence of a functional connection between neurons 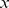 and 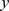, and 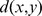 denote the pairwise distance between 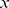 and 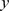. Let 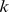 be a pairwise distance. Then,
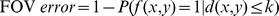
We computed the average FOV error over all pairwise combinations of neurons in all sensory areas as a function of 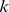. To achieve less than 10 percent FOV error, we found that 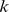 must be at least 676 microns in A1, 660 microns in S1, and 583 microns in V1 (Figure 6C). This corresponds to a minimum of 430 neurons in A1, 510 neurons in S1, and 478 neurons in V1 by computing a cumulative distribution of neuronal density based on the probability distributions of pairwise distances in our fields of view (Figure 6D). In contrast, we found less than 10 percent FOV error was achieved with just 93 microns in 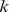-nearest neighbors topologies, and 884 microns (almost the entire imaging field of view) in topologies with a uniform random spatial distribution of functional connectivity (Figure 6C). In the random graphs, error dropped linearly as field of view size was increased (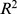 = 0.9995). Thus, it appears that large FOVs result in fewer errors about underlying functional topology, and that the field of view error is lessened by skew in the likelihood of a connection toward shorter distances.

## Discussion

All regions of the sensory neocortex showed a common capacity for spontaneous circuit activations that emerged from the underlying local synaptic connectivity [15]. Using the statistical dependencies of spiking between pairs of neurons, we generated directed and weighted functional graphs. This approach revealed a scaling relationship between A1 and S1 [15], but was unable to delineate exactly what graph features were common to both regions. In this study, we conducted an analysis of graph invariance in functional circuit topologies generated from three regions of sensory neocortex in order to extend the graph theoretic approach toward delineating generalized rules of connectivity. The graph invariant framework allowed us to examine how circuits are similar, by considering how graph properties independent of neuronal labeling are consistent between areas. This represents a top-down approach which extracted global features of functional connectivity from large, dense sampling of neuronal activity in the neocortex. This analysis revealed multiple graph invariants that are consistent across sensory areas. The structure of neocortical functional topologies were well-characterized by non-random connectivity that was not merely dependent on spatial proximity, despite the fact that the probability of functional connection peaked proximally. In all areas, distal connections were required to achieve connected graphs, reminiscent of the daisy arrangement of dense local and patchy distal neocortical connections suggested by neuronal anatomy [2], [48]. We found that functional topologies of all areas were connected, and the degree of connectivity was statistically indistinguishable between areas. Moreover, functional connections were structured even within a local circuit of the functional topology. We found that eigenvector centrality, a measure of influence in local flow, is log-normally distributed in all sensory areas, and is highly correlated with out-degree, and weakly correlated with in-degree. The size of functional topology does not scale with the number of neurons in the field of view, revealing that circuit activity is comprised of structured activations of subsets of neurons. Local circuit flow comprehensively covers angular space regardless of spatial scale, which is inconsistent with a canonical flow of spontaneous activity. Finally, our analysis revealed that given a large imaged field of view, a minimal numerical sample size was necessary to minimize the error of falsely characterizing two neurons as being independent. In summary, the invariant features revealed by this study suggest the existence of a generalized functional circuit throughout the sensory neocortex, strengthening the argument that the neocortical microcircuit hypothesis should be framed as probabilistic rules of connectivity and organization.

This is not to say that label-dependent features do not play a role in mediating the structure of functional topology. For example, although connectivity is strongly biased towards spatial proximity between neurons, the 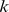-nearest neighbors rule and random topologies poorly recapitulated functional topologies in the data. This indicates that other connectivity rules that are not simply dependent on spatial proximity, such as those based on cell types [11], [49], likely play an important role. As another example, we found that the distribution of eigenvector centrality, which strongly correlates with out-degree in all areas, is highest in V1, and that the ratio of the number of open sequences to closed sequences, which stays constant as a function of path length in all areas, is highest in V1. These analyses suggest that V1 may be more feedforward than A1 and S1, a result consistent with previous studies [37], [42]. The translation of the eigenvector centrality distribution seen in Figure 4A may represent a tweaking of a generalized rule (fitting to a log-normal distribution) to optimize the circuit for a particular function (feedforwardness). In general, it is possible that the specialization of the circuit to the overall function of the cortical area is label-dependent, or dependent on emergent properties of cell phenotypes. However, despite the fact that label dependent rules of connectivity are likely present, by investigating global features of functional circuit topology that are invariant to the details of individual neurons, we are able to reveal abstract structural rules present in functional wiring in a computationally efficient manner.

We emphasize that our functional approach does not necessarily identify causal connectivity, but rather pairwise correlative dynamics [50]. However, we also note that there is a relationship between structure and function [18]. This relationship is likely enhanced in this study as the high sampling density employed here should dramatically increase the likelihood that a correlation could reflect a causal connection, since the likelihood of a synaptic connection increases with spatial proximity [8].

We consider the slice preparation to be an isolated system that allows us to study the local connectivity that defines cortical microcircuitry and remove the potentially conflating influence of long modulatory and long afferent inputs. This approach allowed us to maximize the imaged field of view and the corresponding numerical sample of neurons. In addition, coronal slices allowed us to examine the potential influence of laminar boundaries on functional circuitry. We found that a field of view of approximately 640 µm is necessary to correctly establish functional dependence between two neurons in the sensory neocortex. This field of view results from having a minimal numerical sampling while having sufficient distal functional connections that are necessary to generate a connected graph. The necessity of distal functional connections that extend beyond layers and columns may indicate that functional circuits represent information from multiple octaves in A1 [51] whiskers in S1 [52], or a natural visual scene in V1 [53]. Our data are consistent with anatomical studies that have revealed a patchy, distributed axonal structure which has been postulated to limit signal redundancy while enabling the potential for integration of information within local populations of neurons [48], [54]. For these hypotheses to be properly evaluated, future work toward understanding the role of connectivity in cortical dynamics and behavior will require a combination of research at the *in-vitro* and *in-vivo* level.

Interestingly, we found that the connectedness of the topology depended not only on the size of the field of view, but also on whether the most unreliable connections were considered. In a previous study employing a network model, we similarly found that weak connections were necessary to recapitulate experimentally observed circuit dynamics [15]. In this study, functional topologies became sparse and modular as minimum thresholds on weight were increased, likely because fewer functional connections were reliable. When only the most reliable functional connections were considered, the topologies were sparsely connected regardless of sensory area. By investigating invariant metrics without setting thresholds on how reliably active the neurons were, we did not bias ourselves to only investigating the most reliable connections. Such a bias may lead to subsampling errors, exactly parallel to the problems that arise from using small fields of view. Since circuit topologies become highly connected with the inclusion of weak functional connections, weak connections may be necessary to provide a large dynamic range similar to a previous study of mouse V1 [37], [42]. These data and analyses suggest that the generalized features of functional circuitry identified in this study maximize the capacity of this system to represent the sensory environment.
